# Supplementary material for: Combined Computational–Biochemical Approach Offers an Accelerated Path to Membrane Protein Solubilization
Source: J Chem Inf Model. 2023 Nov 8;63(22):7159–70. doi: 10.1021/acs.jcim.3c00917 (PMC10685452; doi:10.1021/acs.jcim.3c00917)
Supplement: Supplementary file 1 — ci3c00917_si_001.pdf [file ci3c00917_si_001.pdf]

Supporting Information for

A combined computational-biochemical approach offers an accelerated path to membrane protein solubilization

Mariah R. Pierce,[1,‡] Jingjing Ji,[2,‡] Sadie X. Novak,[1] Michelle A. Sieburg,[1] Shivangi Nangia,[3] Shikha Nangia,[2,4,]* and James L. Hougland[1,4,5]*

[1] Department of Chemistry, Syracuse University, Syracuse, NY 13244, USA

[2] Department of Biomedical and Chemical Engineering, Syracuse University, Syracuse, NY 13244, USA

[3] Department of Chemistry, University of Hartford, West Hartford, CT 06117, USA

[4] BioInspired Syracuse, Syracuse, NY 13244, USA

[5] Department of Biology, Syracuse University, Syracuse, NY 13244, USA

‡ These authors contributed equally to this work

*To whom correspondence should be addressed:

Email: snangia@syr.edu, hougland@syr.edu

Phone: (315)443-571, (315) 443-1134

Supporting Figure S1. MEGA-9, LMNG, and Fos-choline family expansions do not improve solubilization.

Supporting Figure S2. MEGA-9, LMNG, and Fos-choline family expansions showed minimal improvement in hGOAT-eGFP activity.

Supporting Figure S3. Convergence analysis for detergent-membrane systems.

Supporting Figure S4. Convergence of RMSF of hGOAT residues in DDM within simulation time course confirms well-equilibrated system.

Supporting Figure S5. Convergence of RMSF of hGOAT residues in BOG within simulation time course confirms well-equilibrated system.

Supporting Figure S6. Convergence of RMSF of hGOAT residues in MEGA-9 within simulation time course confirms well-equilibrated system.

Supporting Figure S7. Convergence of RMSF of hGOAT residues in LMNG within simulation time course confirms well-equilibrated system.

Supporting Figure S8. Convergence of RMSF of hGOAT residues in FOS-16 within simulation time course confirms well-equilibrated system.

Supporting Figure S9. Convergence of RMSF of hGOAT residues in GDN within simulation time course confirms well-equilibrated system.

Supporting Figure S10. Convergence of RMSF of hGOAT residues in FOS-12 within simulation time course confirms well-equilibrated system.

Supporting Figure S11. Convergence of RMSF of hGOAT residues in CHAPS within simulation time course confirms well-equilibrated system.

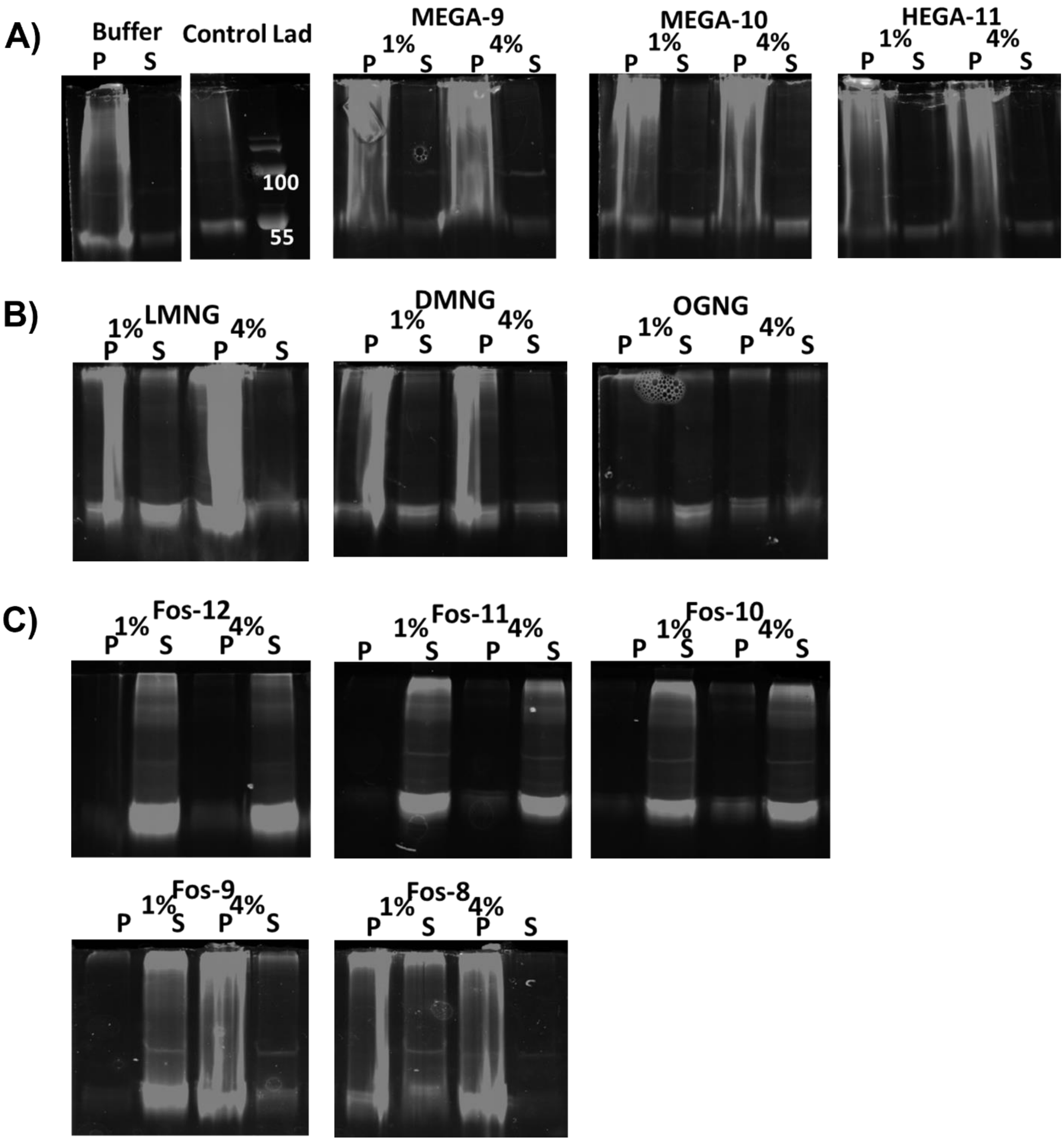

Supporting Figure S1. MEGA-9, LMNG, and Fos-choline family expansions do not improve solubilization. A solubilization screening was completed with the expanded MEGA-9, LMNG, and Fos-choline families. Buffer control is identical to that performed for the detergent

solubilizations in the main text. A) MEGA-9 had comparable solubilization to the detergent screen and MEGA-10 did not improve the solubilization. HEGA-11 depicted does not show solubilization in either detergent percentage. B) DMNG showed the majority of the solubilization in the pellet fraction with some hGOAT-eGFP in the supernatant fractions. OGNG shows partial solubilization, but is not improved from the solubilization from LMNG. C) Fos-12 repeated its solubilization pattern from the initial screen, and the foc-choline detergents with shorter alkyl chain lengths show a gradual decrease in hGOAT-eGFP solubilization.

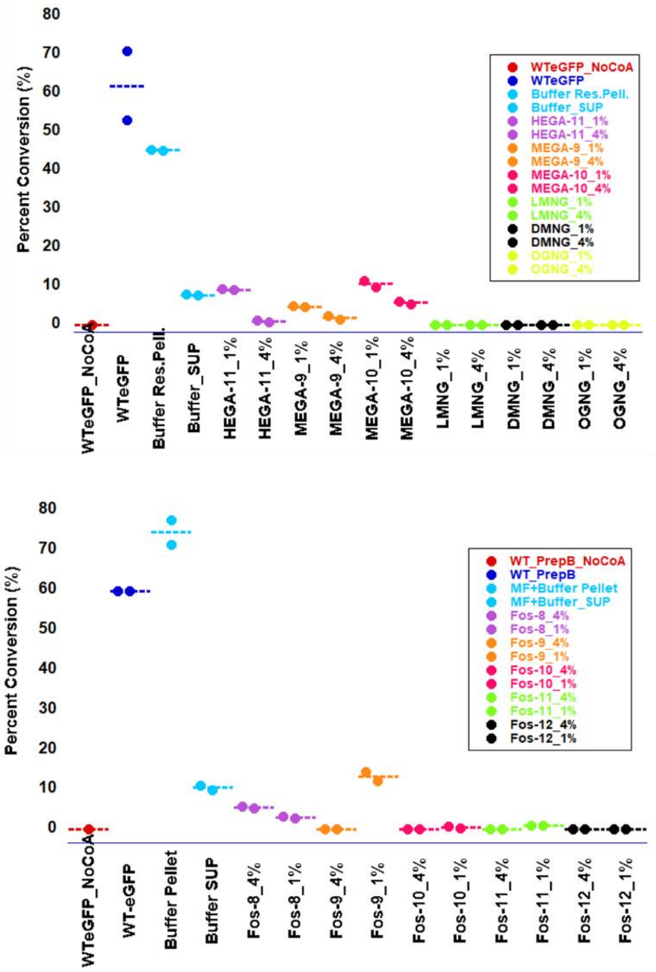

Supporting Figure S2. MEGA-9, LMNG, and Fos-choline family expansions showed minimal improvement in hGOAT-eGFP activity. Supernatant fractions of the solubilization reactions tested for ghrelin acylation activity as described in the main text, with negative and positive controls. hGOAT-eGFP solubilized by HEGA-11, MEGA-10, and MEGA-9 all show similar reactivity to the sample without detergent whereas LMNG, DMNG, and OGNG have inhibited hGOAT-eGFP activity. Amongst the fos-choline detergents, only FOS-9 solubilized hGOAT-eGFP exhibited appreciable acylation activity. Activity screening reactions were performed in duplicate and analyzed as described in Experimental Methods.

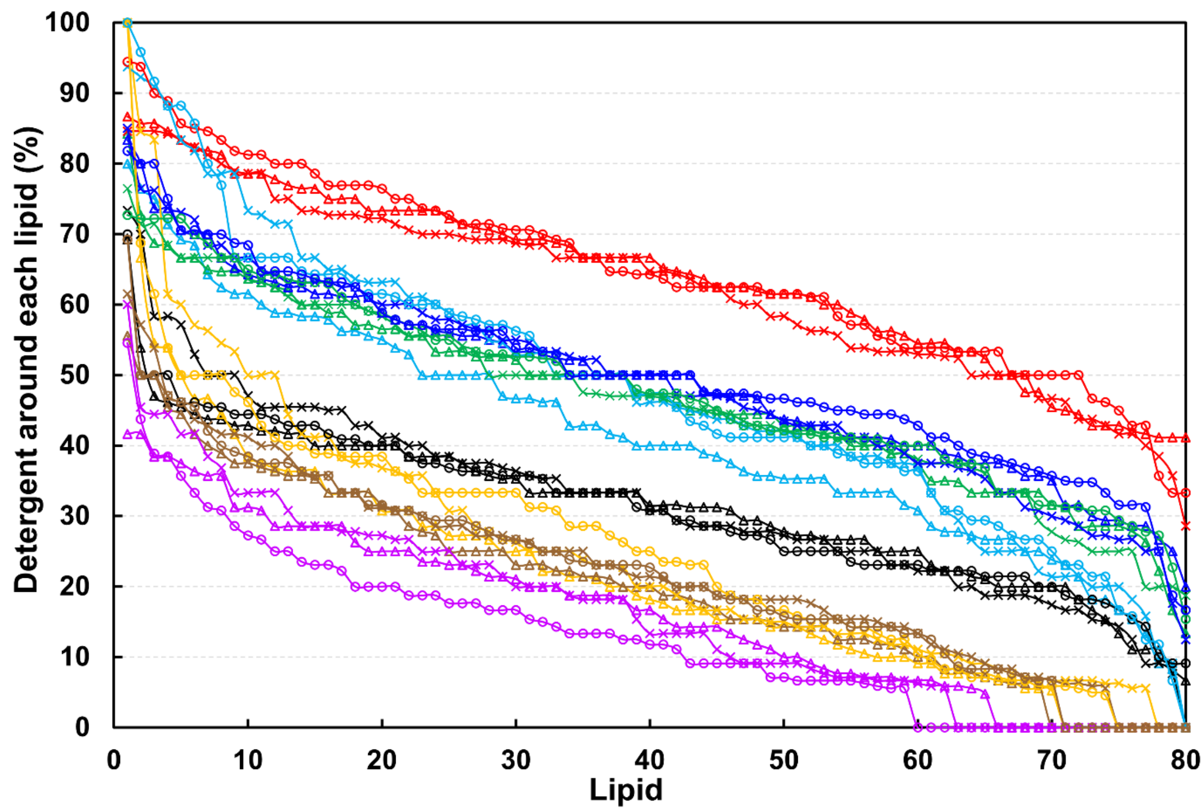

Supporting Figure S3. Convergence analysis for detergent-membrane systems. Percent detergent around each lipid, ranked from highest to lowest population DDM (red), BOG (blue), MEGA-9 (green), LMNG (cyan), FOS-16 (black), GDN (yellow), FOS-12 (brown), and CHAPS (pink). For each detergent-membrane system data is shown for three timepoints during the simulation—1000 ns (triangle), 1500 ns (cross), and 2000 ns (circle).

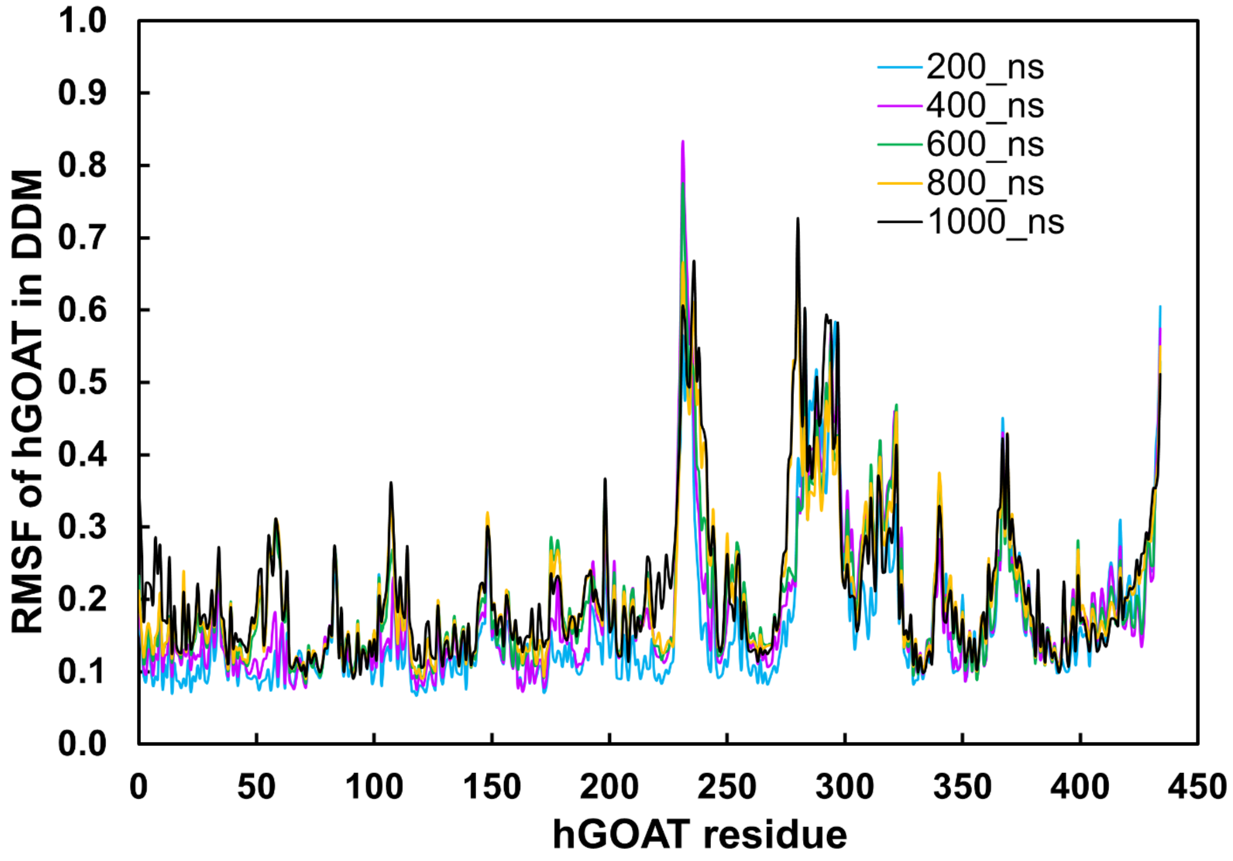

Supporting Figure S4. Convergence of RMSF of hGOAT residues in DDM within simulation time course confirms well-equilibrated system. Root mean square fluctuation (RMSF) of hGOAT residues for DDM at 200 (cyan), 400 (purple), 600 (green), 800 (yellow), and 1000 (black) ns.

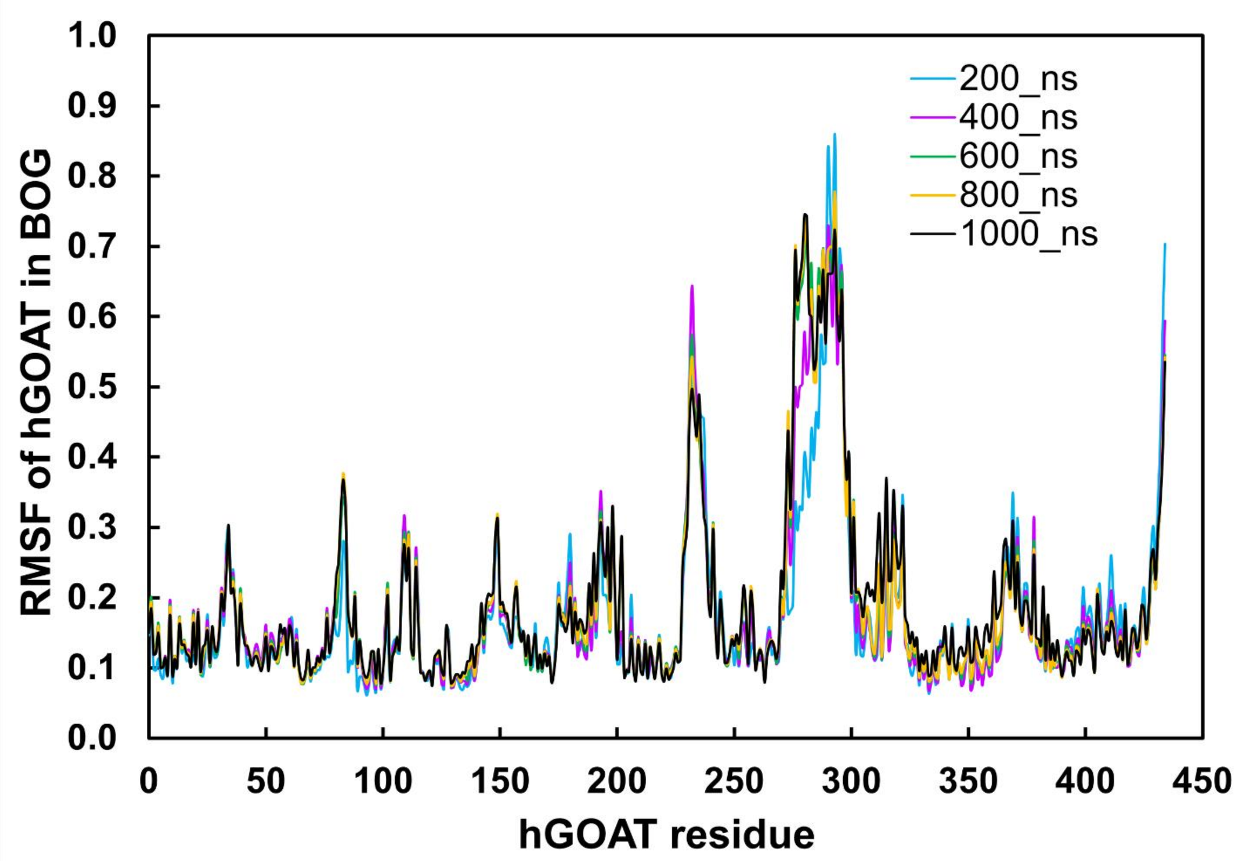

Supporting Figure S5. Convergence of RMSF of hGOAT residues in BOG within simulation time course confirms well-equilibrated system. Root mean square fluctuation (RMSF) of hGOAT residues for BOG at 200 (cyan), 400 (purple), 600 (green), 800 (yellow), and 1000 (black) ns.

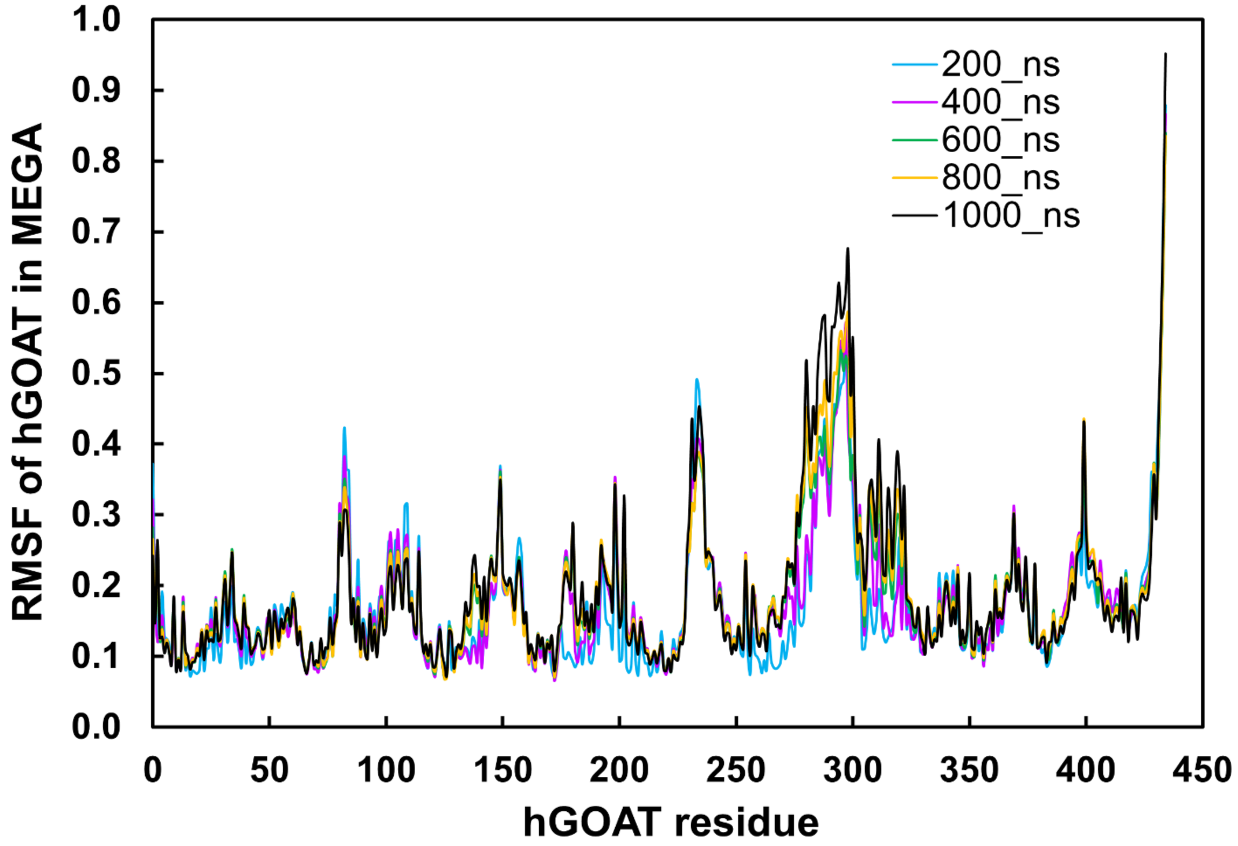

Supporting Figure S6. Convergence of RMSF of hGOAT residues in MEGA-9 within simulation time course confirms well-equilibrated system. Root mean square fluctuation (RMSF) of hGOAT residues for MEGA-9 at 200 (cyan), 400 (purple), 600 (green), 800 (yellow), and 1000 (black) ns.

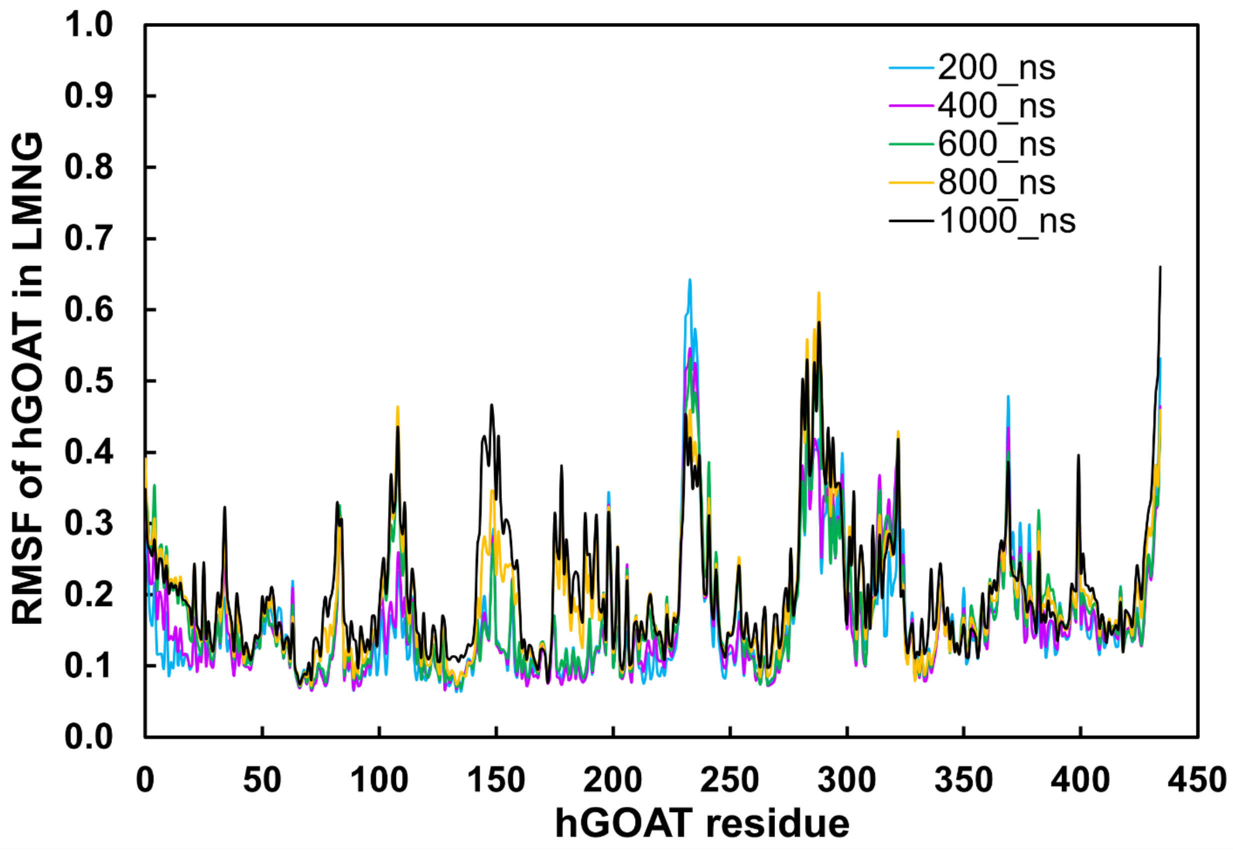

Supporting Figure S7. Convergence of RMSF of hGOAT residues in LMNG within simulation time course confirms well-equilibrated system. Root mean square fluctuation (RMSF) of hGOAT residues for LMNG at 200 (cyan), 400 (purple), 600 (green), 800 (yellow), and 1000 (black) ns.

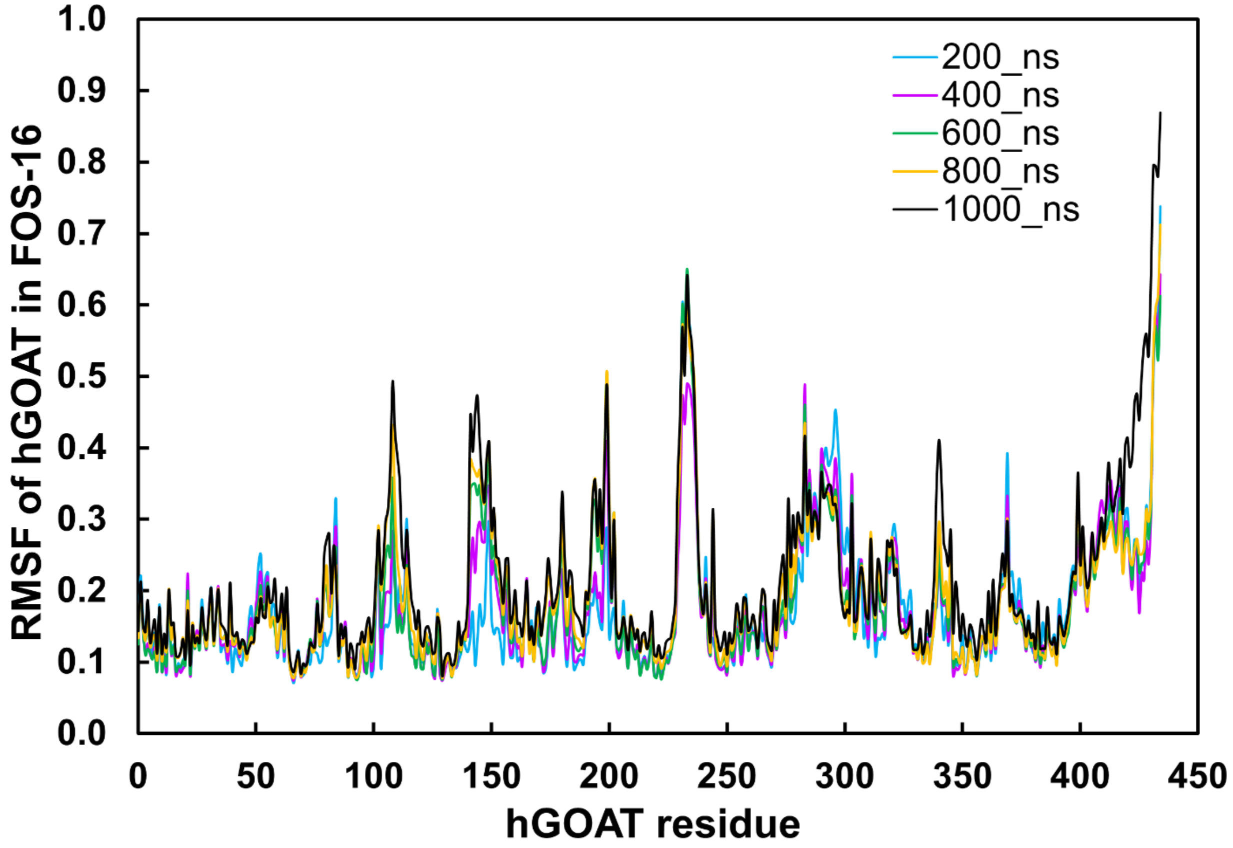

Supporting Figure S8. Convergence of RMSF of hGOAT residues in FOS-16 within simulation time course confirms well-equilibrated system. Root mean square fluctuation (RMSF) of hGOAT residues for FOS-16 at 200 (cyan), 400 (purple), 600 (green), 800 (yellow), and 1000 (black) ns.

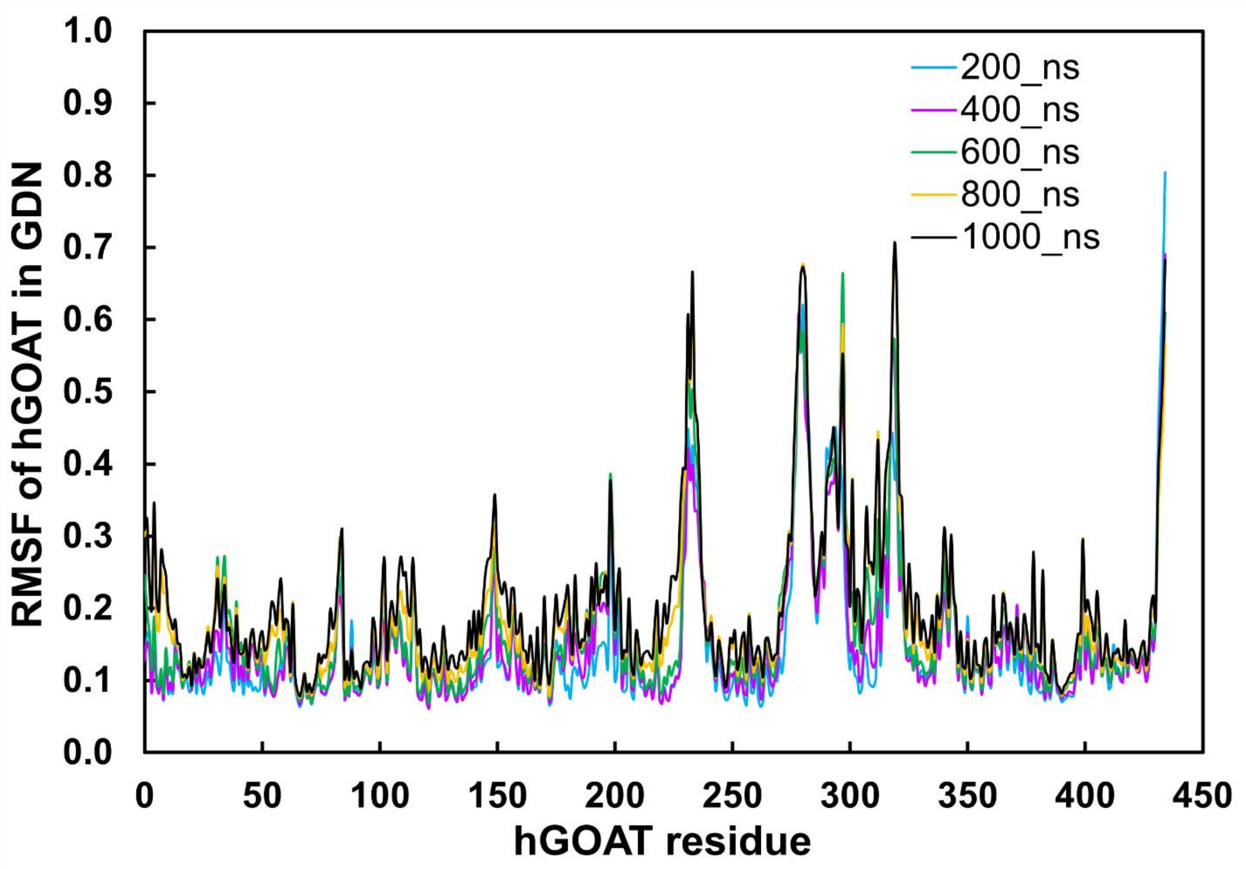

Supporting Figure S9. Convergence of RMSF of hGOAT residues in GDN within simulation time course confirms well-equilibrated system. Root mean square fluctuation (RMSF) of hGOAT residues for GDN at 200 (cyan), 400 (purple), 600 (green), 800 (yellow), and 1000 (black) ns.

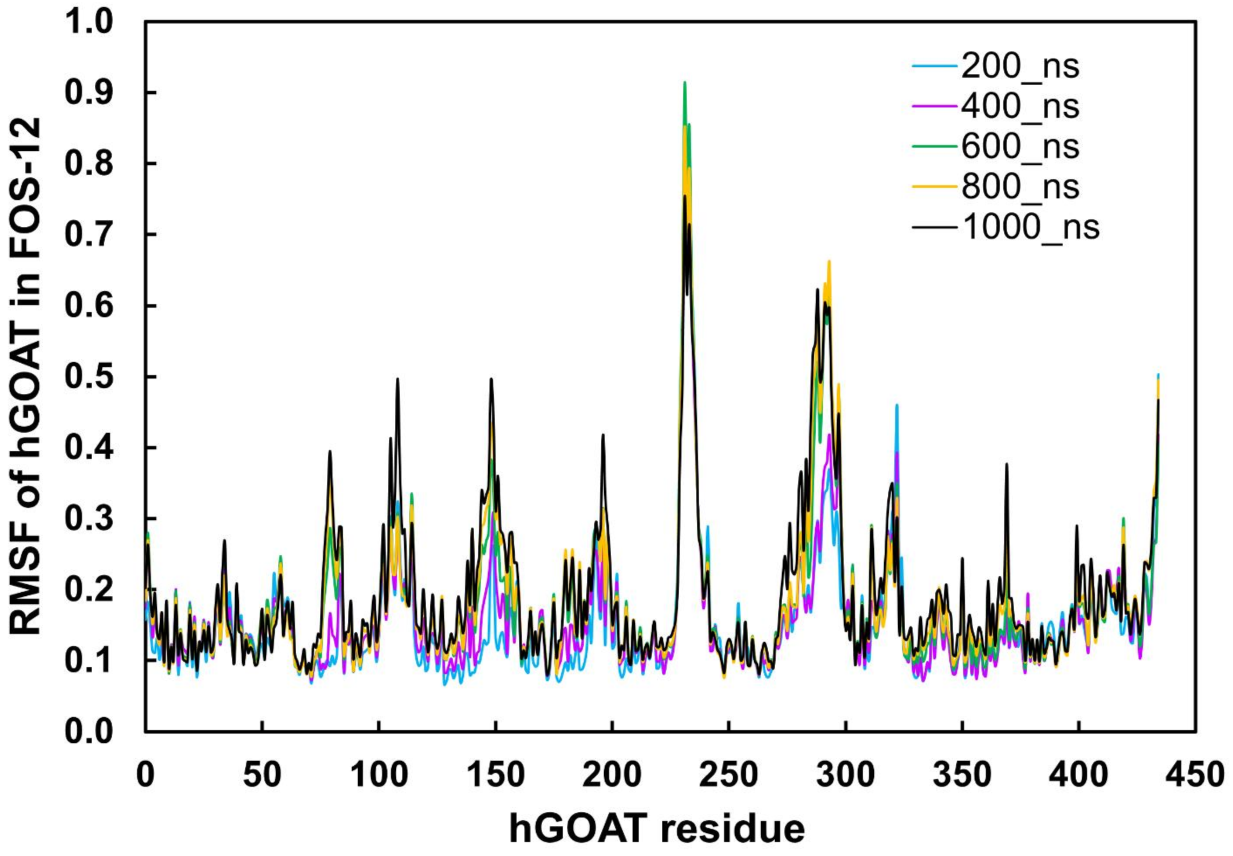

Supporting Figure S10. Convergence of RMSF of hGOAT residues in FOS-12 within simulation time course confirms well-equilibrated system. Root mean square fluctuation (RMSF) of hGOAT residues for FOS-12 at 200 (cyan), 400 (purple), 600 (green), 800 (yellow), and 1000 (black) ns.

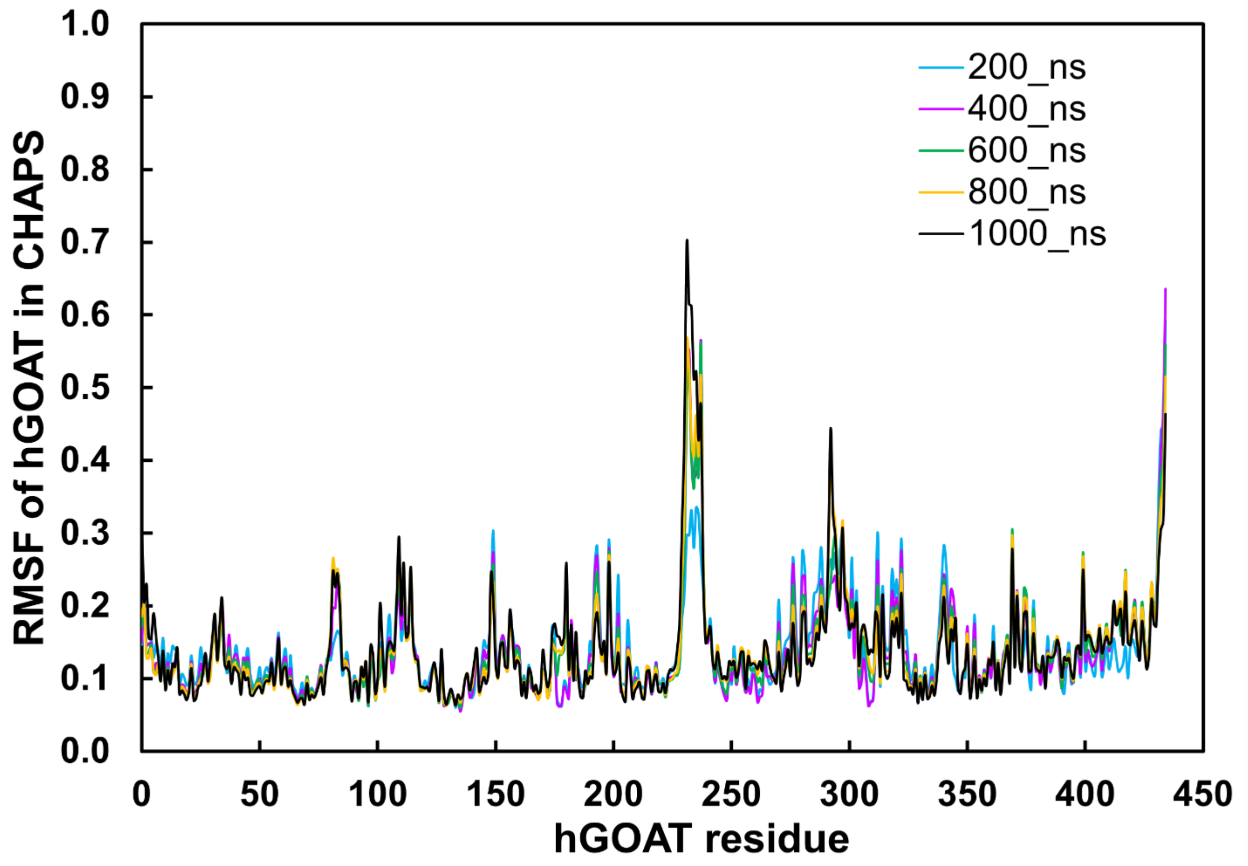

Supporting Figure S11. Convergence of RMSF of hGOAT residues in CHAPS within simulation time course confirms well-equilibrated system. Root mean square fluctuation (RMSF) of hGOAT residues for CHAPS at 200 (cyan), 400 (purple), 600 (green), 800 (yellow), and 1000 (black) ns.
